# Supplementary material for: Identifying rheological regimes within pyroclastic density currents
Source: Nat Commun. 2024 May 23;15:4401. doi: 10.1038/s41467-024-48612-7 (PMC11116420; doi:10.1038/s41467-024-48612-7)
Supplement: Supplementary file 1 — Supplementary Information [file 41467_2024_48612_MOESM1_ESM.pdf]

Supplementary Information to Accompany:

Identifying rheological regimes within pyroclastic density currents

Thomas. J. Jones, Abhishek Shetty, Caitlin Chalk, Josef Dufek and Helge M. Gonnermann

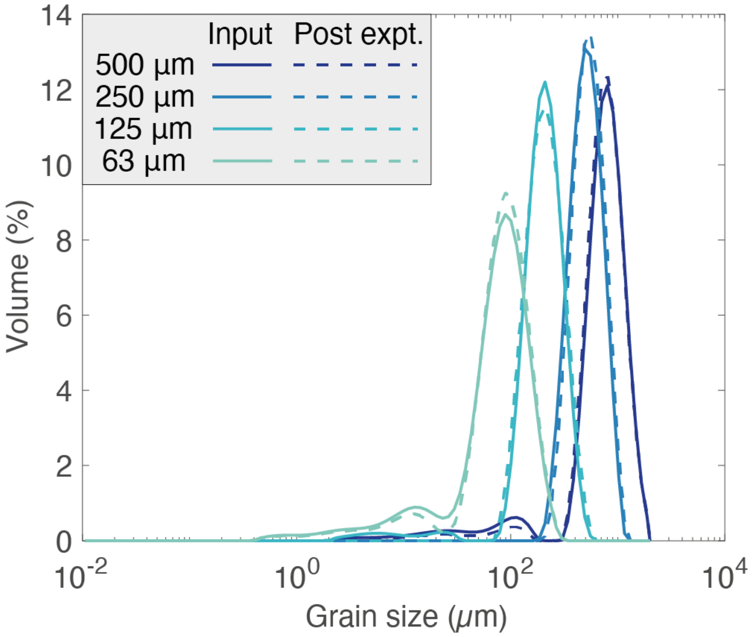

Figure S1: Grain size distribution data for all samples used in this study. Data are colour coded for nominal grain size. The solid and dashed lines represent the material before and after fluidization during the rheometry experiments respectively. No change in grain size distribution is observed allowing us to conclude that no significant particle sized reduction occurred during our rheometry experiments.

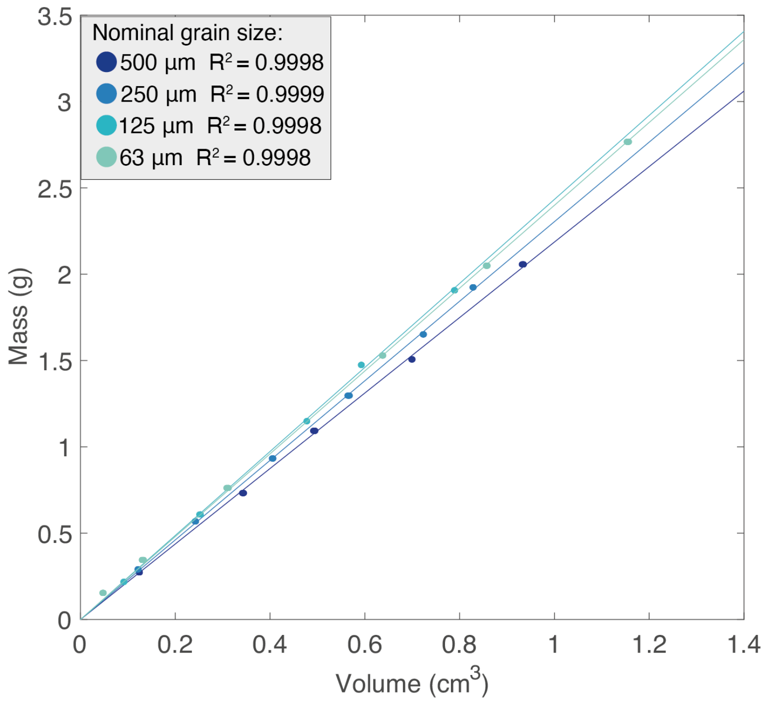

Figure S2: Helium pycnometry data for Mt Meager ash samples plotted as a function of volume (cm^3) and mass (g). Each mass has ten volume measurements. The lines show linear regressions in the form of mass = $\rho_p \times$ volume, where ρ_p is the particle density. The variance (R^2) associated with these fits are reported in the figure legend.

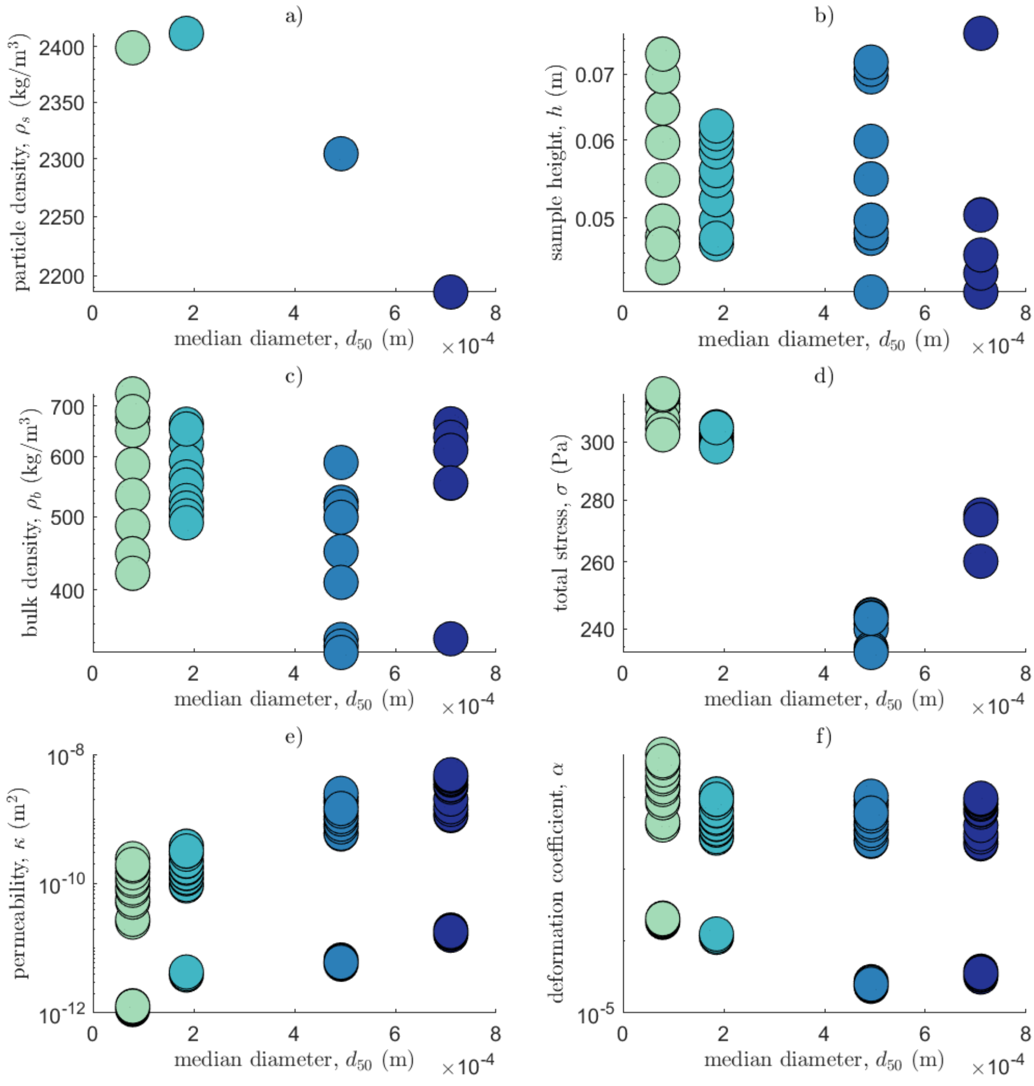

Figure S3: A breakdown of the parameters that compose the dimensionless numbers (Equations 1-3), for each grain size sample: **(a)** particle density, **(b)** sample height, **(c)** bulk density, **(d)** total stress, **(d)** permeability, **(e)** deformation coefficient.

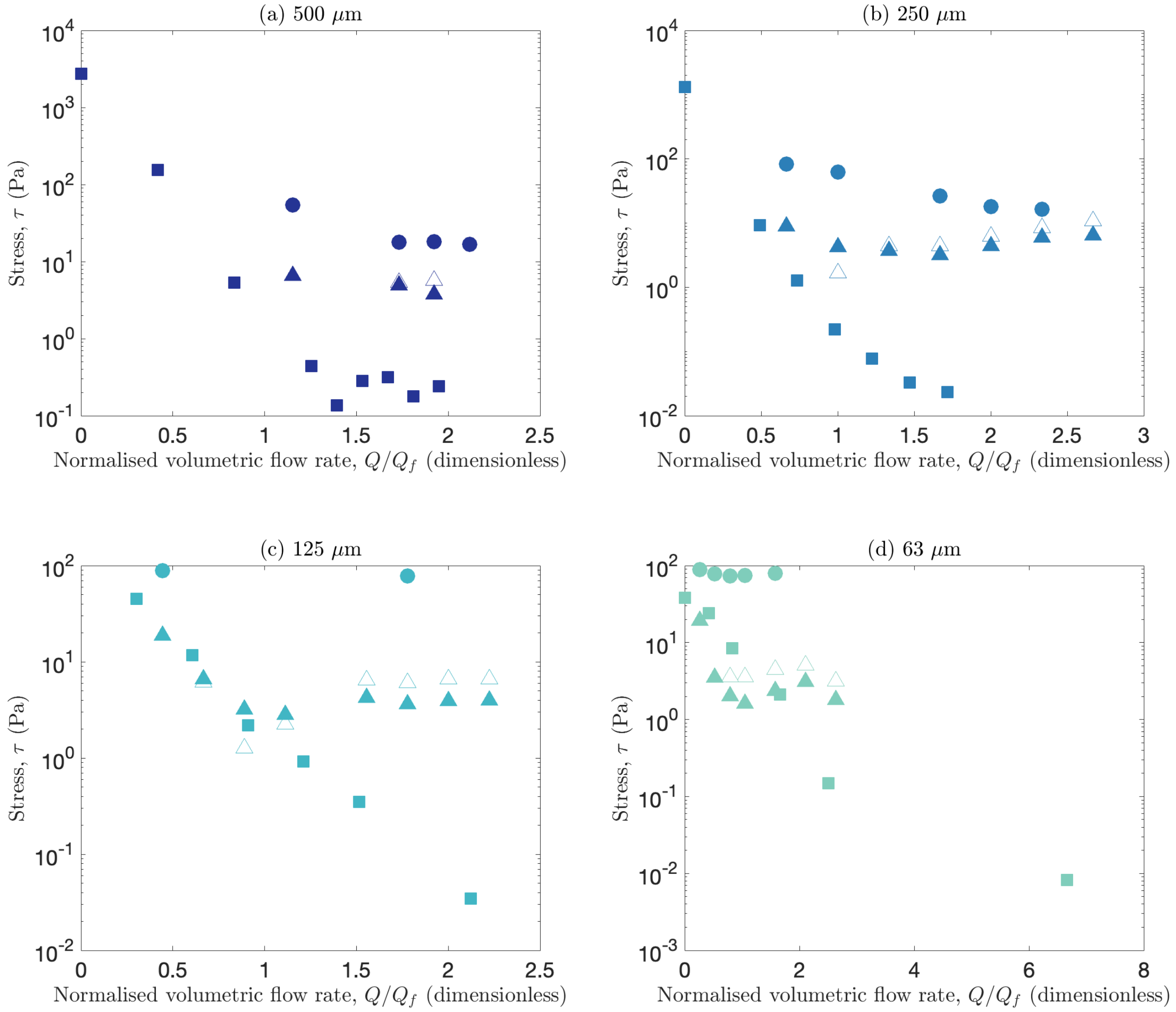

Figure S4: Regime diagrams for the different grain size samples: **(a)** 500 µm, **(b)** 250 µm, **(c)** 125 µm, and **(d)** 63 µm. The squares mark the yield stress and the upper limit of the jammed regime where the granular suspension is locked and immobile. The upwards pointing and sideways pointing triangles mark the measured and modelled lower limit to the shear-thickening regime. The circles mark the measured upper limit to the shear-thickening regime.

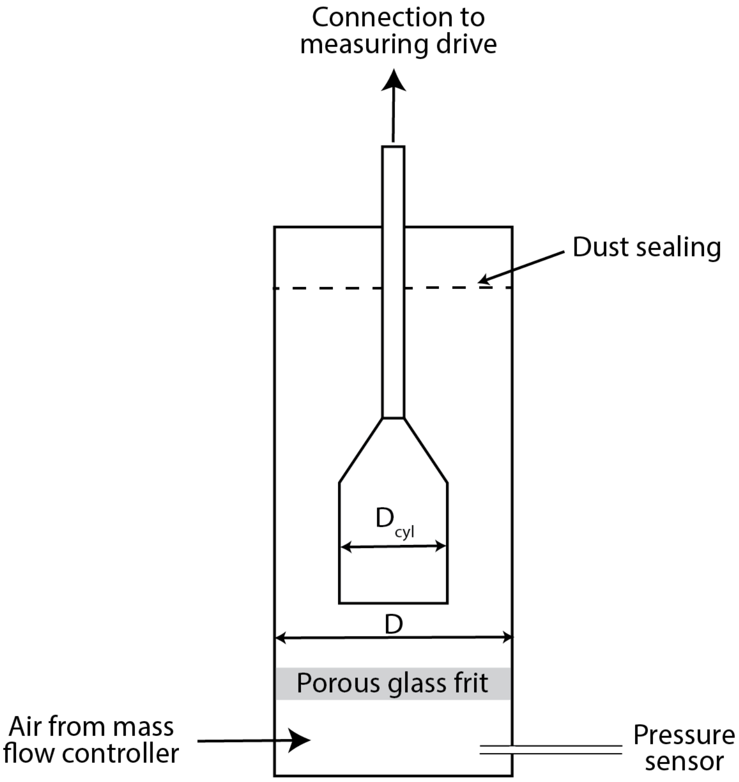

Figure S5: Schematic cross section through the Anton Parr powder flow cell used in this study. The cylindrical section of the measuring geometry, d_{cyl} measures 24.16 mm in diameter, and is housed within the flow cell that has an internal diameter, D of 50 mm.

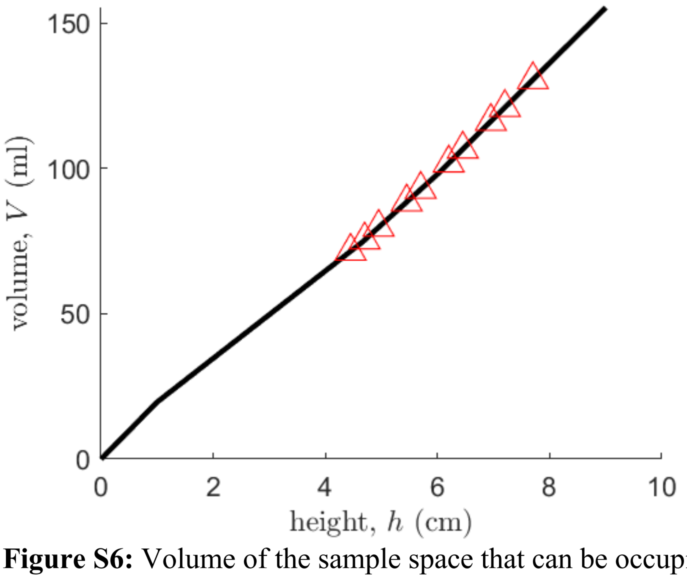

Figure S6: Volume of the sample space that can be occupied as a function of height within the powder flow cell. The red triangles denote the measured values. The black line is the calculated values using the geometry shown in Figure S5.

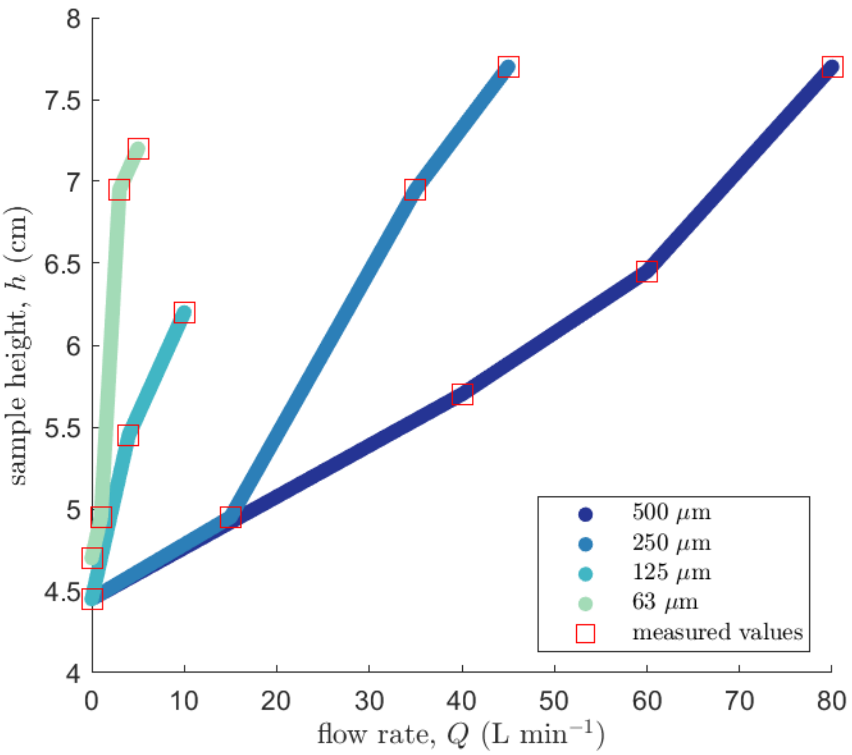

Figure S7: Interpolated sample heights for all flow rates. The red squares denote the measured values provided in Table S2.

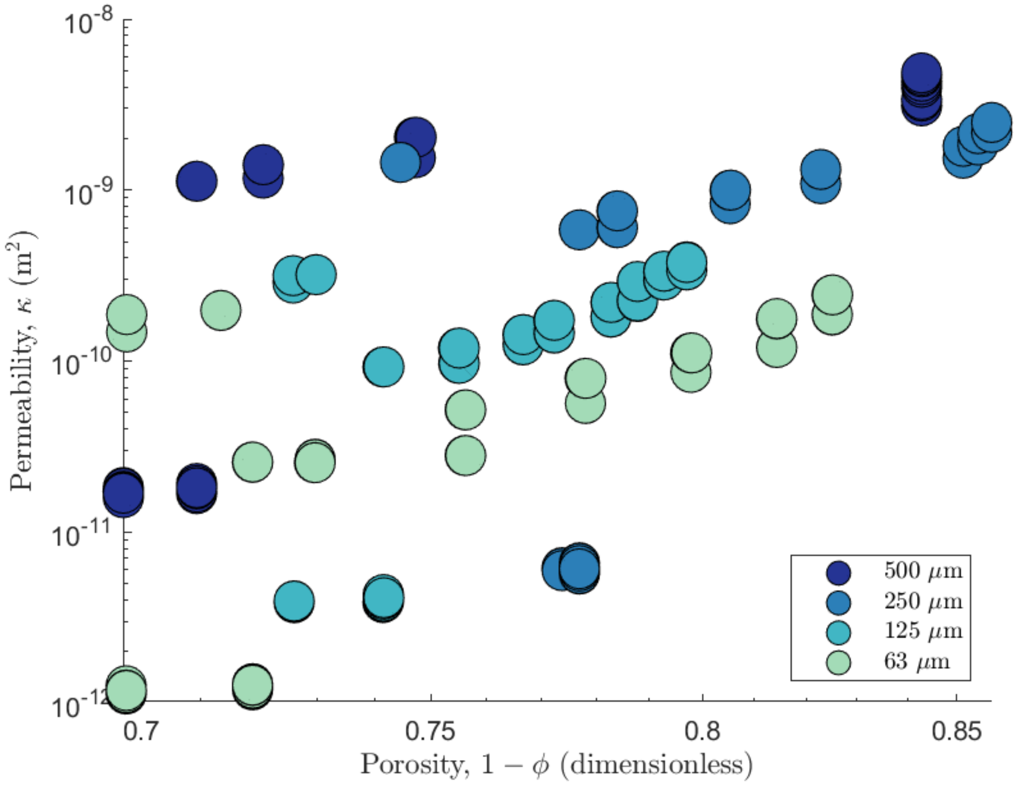

Figure S8: The relationship between porosity and permeability for each sample. Porosities were calculated as described in the Methods. The sample permeability was estimated using Darcy's law.

d [μm]	m [g]	Q [L/min]	h [cm]	V [ml]	ρ_b[kg/m^3]	ϕ	
500	45	0	4.45	71.56	628.85	0.29	Static
250	40	0	4.45	71.56	558.98	0.24	
125	50	0	4.45	71.56	698.72	0.29	
63	52	0	4.70	75.44	689.28	0.29	
500	45	40	5.70	92.87	484.52	0.22	Medium flow rate
250	40	15	4.95	79.80	501.26	0.22	
125	50	4	5.45	88.52	564.87	0.23	
63	52	1	4.95	79.80	651.63	0.27	
500	45	60	6.45	106.71	421.69	0.19	High flow rate
250	40	35	6.95	116.28	344.00	0.15	
125	50	10	6.20	101.93	490.53	0.20	
63	52	3	6.95	116.28	447.20	0.15	
500	45	80	7.70	130.63	344.49	0.14	Max flow rate
250	40	45	7.70	130.63	429.49	0.18	
125	50	10	6.20	101.93	490.53	0.20	
63	52	10	7.20	121.11	306.21	0.13	

Table S1: Sample heights h, volumes of particles V occupying the powder cell, calculated bulk density ρ_b, and particle volume fraction ϕ, for all grain sizes, d. at rest, medium and high volumetric flow rates. These particle volumes account for the volume occupied by the measuring system and are reported for static (i.e., 0 L min^{-1}), medium, high, and maximum volumetric flow rates, Q.

d [µm]	Q [L/min]	τ [Pa]
500	0	2681.8
500	15	147.4
500	30	40.272
500	45	14.351
500	50	13.629
500	55	15.309
500	60	0.5907
500	65	0.39343
500	70	0.63646
250	0	1276.3
250	10	61.58
250	15	30.592
250	20	21.123
250	25	19.078
250	30	16.279
250	35	13.557
250	40	0.012278
125	0	424.64
125	1	47.234
125	2	70.775
125	3	2.6644
125	4	35.166
125	5	37.764
125	7	35.795
125	8	41.077
125	9	37.52
125	10	35.626
63	0	54.297
63	0.25	29.166
63	0.5	72.922
63	1	39.477
63	1.5	51.719
63	2	45.911
63	3	46.34
63	4	48.305
63	5	0.0023171

Table S2: The shear stresses above which the rheology data were no longer considered during fitting of Equation 4.
