# Supplementary material for: Identifying rheological regimes within pyroclastic density currents
Source: Nat Commun. 2024 May 23;15:4401. doi: 10.1038/s41467-024-48612-7 (PMC11116420; doi:10.1038/s41467-024-48612-7)
Supplement: Supplementary file 3 — Description of Additional Supplementary Files [file 41467_2024_48612_MOESM3_ESM.pdf]

Description of Additional Supplementary Files

File Name: Supplementary Data 1
Description: An excel file containing all the raw pressure drop and air flow rate data in Figure 1. The file has four tabs, each corresponding to a nominal grain size (500, 250, 125 and 63 microns).

File Name: Supplementary Data 2
Description: An excel file containing all the rheology data in Figure 3. The file has four tabs, each corresponding to a nominal grain size (500, 250, 125 and 63 microns).
